# Morphological and Morphometric Relations of Infraorbital Foramen in North Indian Population

**DOI:** 10.7759/cureus.34525

**Published:** 2023-02-01

**Authors:** Arpita Mahajan, Ranjana Verma, Shayama K Razdan, Jigyasa Passey

**Affiliations:** 1 Anatomy, Hamdard Institute of Medical Sciences and Research, New Delhi, IND; 2 Anatomy, Government Institute of Medical Sciences​, Greater Noida, IND; 3 Anatomy, Vardhman Mahavir Medical College and Safdarjung Hospital, New Delhi, IND

**Keywords:** infraorbital nerve, infraorbital canal, skull, anatomy, infraorbital foramen

## Abstract

Introduction

The evidence regarding the anatomy of the infraorbital foramen in the Indian population is limited. It mainly focuses on its shape, size, and incidence in the Indian population. The current study aimed to evaluate morphometric parameters of infraorbital foramen that can help clinicians during surgery and procedures around it.

Methods

We evaluated 90 dry adult human hemi-skulls. The morphological parameters studied included the assessment of the shape of the infraorbital foramen, its horizontal and vertical diameters, and its relation to the teeth of the upper jaw. In addition, we measured the distance of the infraorbital foramen from the anterior nasal spine, nasion, infraorbital margin, and the lower extent of the alveolar margin. The length of the infraorbital canal till the inferior orbital fissure and the infraorbital groove and the infraorbital canal orientation angles in different planes were also measured. The measurement values were compared between the right and left side hemi-skulls.

Results

The oval-shaped infraorbital foramen was most commonly noticed. The mean vertical and transverse diameters were 3.8 mm and 2.6 mm, respectively, on the right side. The left side's mean vertical and transverse diameters were 3.9 mm and 2.5 mm, respectively. The most common location of infraorbital foramen was in line with the maxillary second premolar tooth. The distances of infraorbital foramen from the alveolar margin were 29.6 mm and 29 mm on the right and left sides, respectively. The distances of the infraorbital foramen from the anterior nasal spine were 34.3 mm and 34.2 mm on the right and left sides, respectively. The distances of infraorbital foramen from the nasion were 42.3 mm and 42.2 mm on the right and left sides, respectively. The distances of infraorbital foramen from the inferior orbital margin were 5.8 mm and 6.2 mm on the right and left sides, respectively. The distances between the inferior orbital margin and infraorbital groove were 12.7 mm and 12.7 mm on the right and left sides, respectively. The distances between the inferior orbital margin and inferior orbital fissure were 27.5 mm and 27.1 mm on the right and left sides, respectively. The orientation angles of infraorbital foramen were 48.31° in the horizontal plane, 34.07° in the Frankfurt plane, and 14.4° in the parasagittal plane.

Conclusion

Our findings suggest that the location of the infraorbital foramen is difficult to standardize, considering the wide interindividual variations in the foramen relations. Further research should be performed to investigate the parameters related to the distance and orientation of the infraorbital foramen in relation to nearby bony landmarks that are least affected by individual variations in skull morphology.

## Introduction

The infraorbital nerve is the terminal sensory branch of the maxillary nerve. The infraorbital nerve and the infraorbital artery traverse anteriorly into the orbital floor through the infraorbital groove. They then enter the infraorbital canal and exit through the maxillary bone's infraorbital foramen. Within the infraorbital canal, the infraorbital nerve branches into the anterior-superior and middle-superior alveolar nerves, which supply the incisor and canine teeth, and premolar and first molar teeth, respectively. The nerve is flanked by the levator labii superioris and levator anguli oris muscles before reaching the skin [1]. Further branches of the infraorbital nerve supply the skin and conjunctiva of the inferior eyelid, part of the cheek, part of the nose, the skin and mucosa of the upper lip, the mucosa of the maxillary sinus, some of the upper teeth and adjoining gingivae [2].

The infraorbital neurovascular bundles are important structures that must be taken care of during procedures around the oral and maxillofacial areas. The infraorbital nerve is a sensory nerve that requires anesthetization for operations in dentistry, plastic surgery, and ophthalmology. Blocking this nerve is also useful for treating intractable and pharmacologically unresponsive trigeminal neuralgia [1-3]. The localization of the infraorbital foramen is crucial to avoid clinical complications such as entrapment neuropathies, neuralgias, bleeding, and loss of sensation in corresponding regions of the face [1-3].

Previous studies have reported that for applying an infraorbital nerve block, the surgeon needs to palpate the infraorbital rim to identify the infraorbital foramen and then insert the needle upward to inject the local anesthetic agent [3,4]. It is, therefore, important to know the infraorbital foramen's location and the infraorbital canal's direction.

The studies concerning the infraorbital foramen and infraorbital canal morphology are limited, especially in the North Indian population. Furthermore, most such studies are radiological ones determining the orientation of infraorbital foramen in different planes [5-7]. The current study investigated the morphometric relations of the infraorbital foramen and canal in dry adult human skulls.

## Materials and methods

This observational study was performed on 45 dry human skulls (90 hemi-skulls) of unknown age and sex at our anatomy department after approval from the Institutional Review Board (Research and Project Approval Committee, Hamdard Institute of Medical Sciences and Research, New Delhi, IND, and Approval Number: exempted). The skull specimens were selected after excluding those with fractures and visible physical damage. The sagittal plane for measurements was considered parallel to the mid-sagittal plane but passed through the center of the infraorbital foramen. It was used for measuring various vertical dimensions. A plane passing through the center of the infraorbital foramen and perpendicular to the sagittal plane mentioned above was used for measuring transverse dimensions. All measurements were made on both sides of the skull. We used digital Vernier calipers to measure distance-related measurements.

**Figure 1:**
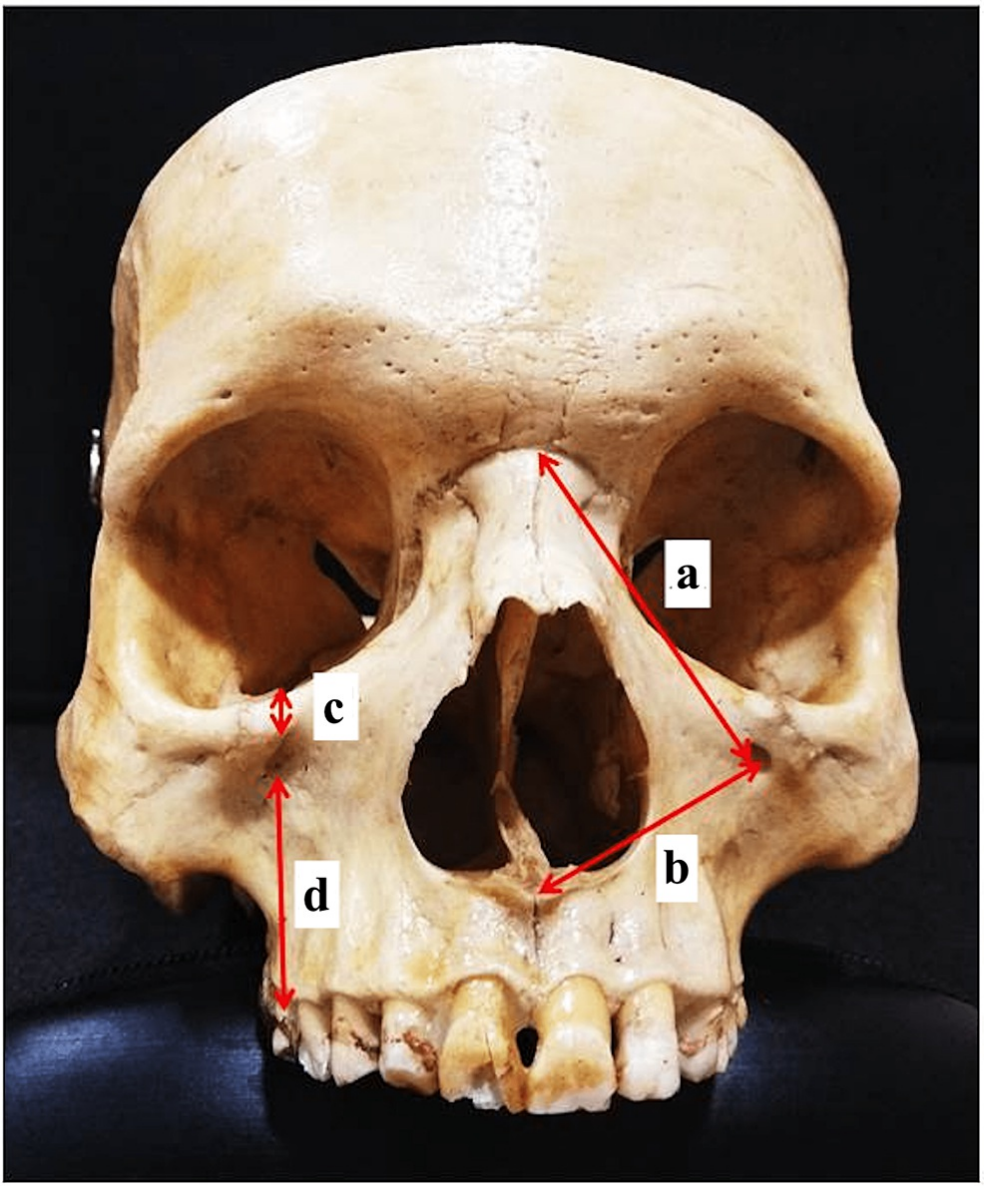
Measurement of the distance of the center of the infraorbital foramen was measured from the nasion (a), anterior nasal spine (b), inferior orbital margin (c), and lower end of the alveolar margin (d).

**Figure 2:**
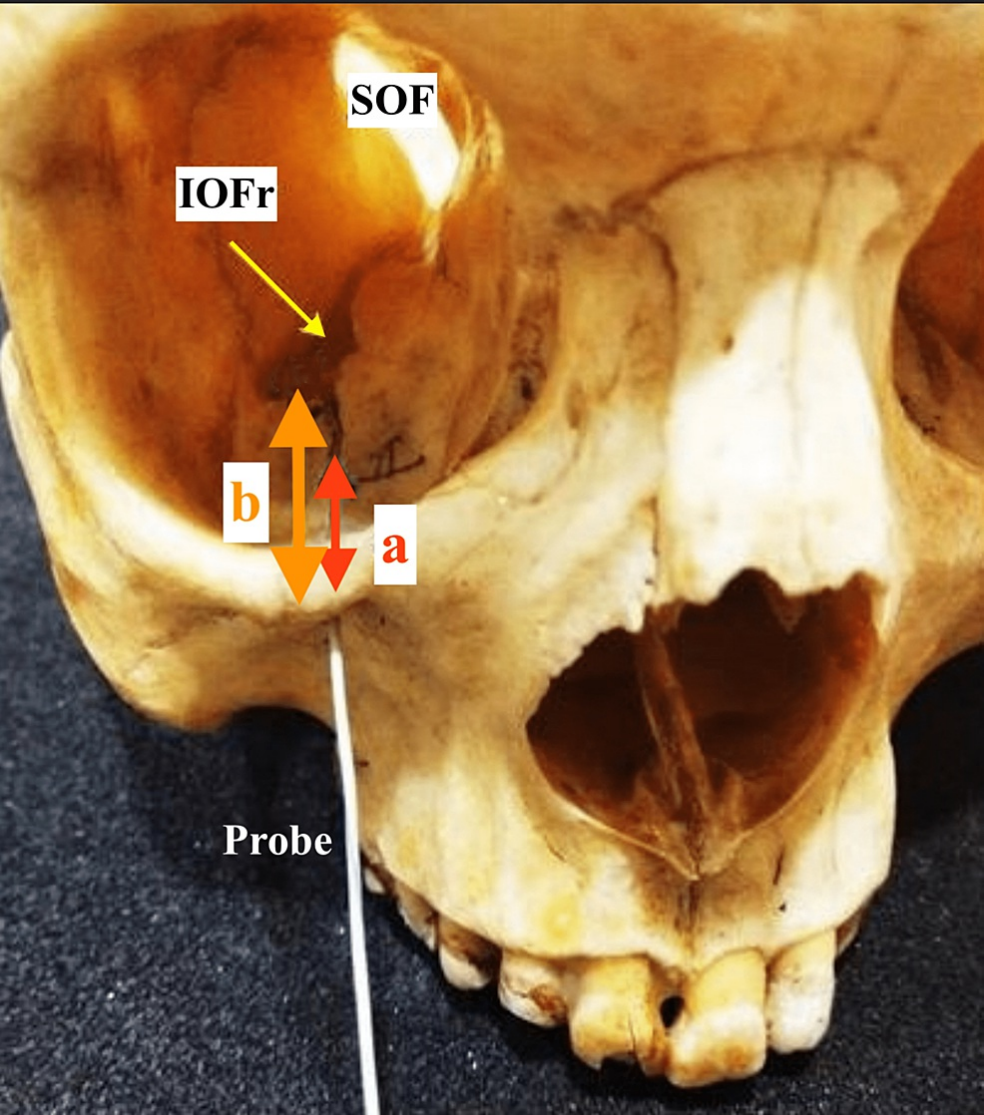
Figure showing the probe inside the right infraorbital foramen. Measurements of the distance of the inferior orbital rim from the anterior part of the infraorbital groove (a) and from the anterior inferior orbital fissure (b) have been shown. IOFr: inferior orbital fissure, SOF: superior orbital fissure

**Figure 3:**
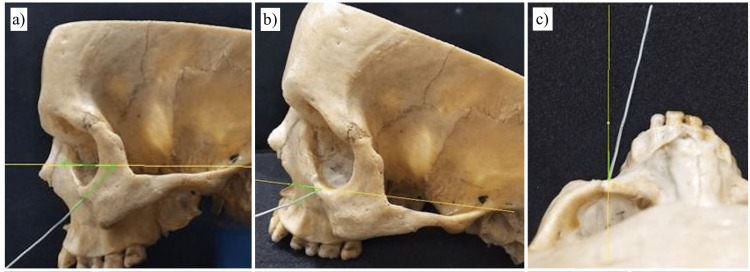
Angular measurements of the infraorbital canal, taken in different planes: (a) entry angle in the horizontal plane, (b) entry angle in the Frankfurt plane, and (c) entry angle in the parasagittal plane.

First, we measured the distance of the center of the infraorbital foramen from the nasion, anterior nasal spine, inferior orbital margin, and lower end of the alveolar margin (Figure 1). The latter two were vertical measurements. Second, we measured the length of the infraorbital canal from the inferior orbital foramen to the inferior orbital fissure's anterior-most extent and to the infraorbital groove's anterior extent in line with the direction of the infraorbital canal. The length was measured using a linear probe directed inside the infraorbital canal (Figure 2). Third, a linear probe was inserted into the infraorbital foramen, and its orientation angles were measured in horizontal, Frankfurt, and parasagittal planes using computer assistance (Figure 3) [8].

Additionally, the skulls were inspected for the shape of infraorbital foramen by direct visual inspection on both sides. Furthermore, the vertical and transverse diameters of the foramen were measured. Two anatomy faculty members with more than 10 years of experience in morphometric measurements made the assessments. Their interobserver intraclass correlation was high for all the measurements made. The means of their measurements were taken as final readings.

The categorical parameters were expressed as percentage/frequency distribution. The continuous measurements were expressed as mean (range). The right and left sides' measurements were compared using the Mann-Whitney U test. A p-value < 0.05 was considered statistically significant.

## Results

**Figure 4:**
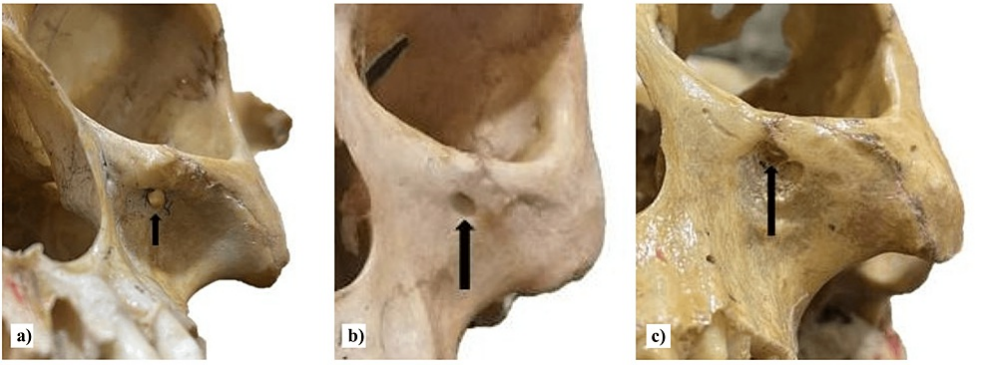
The figure shows the round (a), oval (b), and triangular (c) shapes of the infraorbital foramen.

Among the observed hemi-skulls, the most common shape of the infraorbital foramen was oval (68% on the right side and 60% on the left). The other shapes encountered were round (24% on the right side, 28% on the left) and triangular shapes (8% on the right side and 12% on the left) (Figure 4). The mean vertical diameter was 3.8 mm (1.94-5.94 mm) on the right side and 3.9 mm (2.15-6.06 mm) on the left side. The mean transverse diameter was 2.6 mm (1.5-3.8 mm) on the right side and 2.5 mm (1.08-3.38 mm) on the left side. In most cases, the infraorbital foramen was in line with the second maxillary premolar tooth (44% on the right side and 52% on the left side). The second most common location was in line with the first maxillary molar (32% on the right side and 28% on the left). In the remaining cases (24% on the right side and 20% on the left), the infraorbital foramen was aligned with the second maxillary molar tooth. The were no significant differences between right and left-side observations.

The distance-wise relations of infraorbital foramen from different bony landmarks on the skull have been presented in Table 1. The length of the infraorbital canal from the infraorbital foramen to the infraorbital groove and the inferior orbital fissure is presented in Table 2. The angular measurements related to the orientation of the infraorbital canal are presented in Table 3.

**Table 1:** The distance of infraorbital foramen from different bony landmarks.

S.No	Parameter	Right side measurements (in mm) Mean (range)	Left side measurements (in mm) Mean (range)	P-value
1	Distance of infraorbital foramen from nasion	42.34 (36.17-47.01)	42.2 (38.42-46.45)	0.75
2	Distance of infraorbital foramen from anterior nasal spine	34.36 (30.79-38.72)	34.2 (30.16-37.50)	0.64
3	Verical distance of infraorbital foramen from inferior orbital margin	5.81 (3.23-8.05)	6.25 (3.69-9.41)	0.07
4	Vertical distance of infraorbital foramen from alveolar margin	29.63 (23.50-36.91)	29.4 (21.81-35.54)	0.08

**Table 2:** Length of the infraorbital canal up to different bony landmarks in orbit.

S.No	Parameter	Right side measurements (in mm) Mean (range)	Left side measurements (in mm) Mean (range)	P-value
1	Length of the infraorbital canal from the infraorbital foramen to the anterior part of the inferior orbital groove	12.7 (5.37-21.83)	12.7 (6.56-21.97)	0.89
2	Length of the infraorbital canal from the infraorbital foramen to the inferior orbital fissure	27.5 (22.51-32.19)	27.1 (9.06-35.18)	0.46

**Table 3:** Orientation angles of the infraorbital canal in different planes.

S.No	Parameter	Right (n=45)	Left (n=45)	P-value
1	Entry angle of the infraorbital foramen in the horizontal plane	48.3 (46.4-49.1)	47.8 (45.2-48.3)	0.42
2	Entry angle of the infraorbital foramen in Frankfurt plane	34.5 (33.5-35.2)	33.9 (32.6-34.2)	0.32
3	Entry angle of the infraorbital foramen in the parasagittal plane	15.4 (14.2-15.9)	14.3 (13.9-14.6)	0.89

## Discussion

The present study studied morphology and morphometric relationships of infraorbital foramen. Our findings suggest that the morphometric relations of infraorbital foramen are highly influenced by individual variations in the skull dimensions, evident in the wide ranges of distance-wise measurements presented in Tables 2, 3. Thus, the localization of infraorbital foramen in an individual may not be feasible purely through the morphometric data. Besides clinical evaluation through palpation, additional adjuncts like ultrasound guidance may be helpful for precise localization of the infraorbital foramen. While the distance-based dimensions varied widely, our findings suggest that the infraorbital canal orientation showed only minor variation among the evaluated skulls. Thus, the angular measurement seemed reliable for assessing the direction of the infraorbital canal once the infraorbital foramen is localized.

In the present study, the mean distance of infraorbital foramen from the inferior orbital margin was 5.81 mm on the right and 6.25 mm on the left side, comparable to the findings of Singh et al. [9], Aggarwal et al. [10] in North Indians. It was also comparable to a Brazilian study by Chracanovic et al. [11] and a study by Elsheikh et al. [12] in the Egyptian population. Some south Indian studies showed values (> 7 mm) that were higher than ours [13,14]. Studies on African American skulls by Zang et al. [15] and Nigerian skulls by Ukoha et al. [16] also reported higher values. The ranges of this distance varied between 2 mm and 16 mm in different studies done in India and internationally.

The anterior nasal spine is another commonly used bony landmark to locate the infraorbital foramen. Our study reported mean values of 34.36 mm on the right side and 34.2 mm on the left side, similar to the values reported by Swaminathan et al. [17] in South Indian population, Zang et al. [15] in African American populations, and Lopes et al. [18] in the Brazilian population. However, Ukoha et al. [16] reported a much lower value (~30 mm) in Nigeria.

Distance of infraorbital foramen from nasion is another landmark considered while locating the foramen. The present study reported a value of 42.34 mm on the right side and 42.2 mm on the left side, similar to the study done by Nanayakkara et al. [19] in Sri Lanka. On the other hand, few studies reported slightly higher values (~45 mm). One study was by Singh et al. [9] in the North Indian population, and another by Przygocka et al. [20] in the Polish population. On the contrary, a south Indian study by Ekambaram et al. [13] reported values lower than 40 mm.

Considering the vertical alignment with maxillary teeth, we found that the infraorbital foramen was most commonly aligned with the second upper premolar, consistent with most Indian and International studies [16,21,22]. We also measured the distance of the infraorbital foramen from the alveolar margin, which was 29.6 mm on the right side and 29.4 mm on the left side. These findings were comparable to the studies by Polo et al. [23] conducted in Honolulu and by Aggarwal et al. [10] in North India. However, Swaminathan et al. [17] and Nanayakkara et al. [19] observed lower values (~27 mm) in the South Indian population.

The entry angle of the infraorbital foramen is important for procedures around the infraorbital canal. In the present study, the values were obtained in different planes as 48.31° (right) and 47.31° (left) in the horizontal plane, 34.68° (right), 33.46° (left) in the Frankfurt plane, and 14.18° (right) and 14.61° (left) in the parasagittal plane. Still, the differences in our findings were quite evident compared to the other studies (Table 4). Besides the conventional planes, we used the Frankfurt plane because it closely matches the natural head position [8]. Thus, it makes the measurements clinically relevant. The Frankfurt plane passes through the upper borders of the external auditory meatus, and through the inferior border of the orbital rim [8]. It is widely accepted in cephalometric usage.

**Table 4:** Comparison of the infraorbital canal orientation angles in different planes.

Study	Orientation angle	Plane of measurement
Lee et al. [3]	Right: 13°, left: 11°	Median
	Right: 44°, left: 44°	Frankfurt
Rahman et al. [5]	22°	Coronal
	120°	Sagittal
Aggarwal et al. [10]	Right: 21.48°, left: 20.81°	Sagittal
	Right: 31.43°, left: 32.10°	Frankfurt
Açar et al. [6]	36.57°	Sagittal
	56.80°	Axial
Bahşi et al. [7]	Right: 34.81°, left: 34.44°	Sagittal
Present study	Right: 48.31°, left: 47.31°	Horizontal
	Right: 34.68°, left: 33.46°	Frankfurt
	Right: 14.18°, left: 14.61°	Parasagittal

The shape of infraorbital foramen was oval in most previous studies, and the vertical and transverse diameters were comparable to our findings [9-21]. The length of the infraorbital canal is important, considering that the needle for invasive procedures should not penetrate the orbital floor. However, wide variations have been noticed in the measurements of infraorbital canal length among different studies and when compared to our findings (Table 5).

**Table 5:** Comparison of infraorbital canal length among different studies.

Study	Measurement (mm)	Population
Kazkayasi et al. [24]	22.95	Turkish
Huanmanop et al. [25]	Right: 12.3, left: 12.4	Thai
Rahman et al. [5]	14	Florida
Przygocka et al. [20]	Right: 14.23, left: 13.71	Polish
Nguyen et al. [1]	15.8	USA
Bahsi et al. [7]	Right: 8.28, left: 8.45	Turkey
Present study	Right: 12.7, left: 12.7	North Indian

Concerning the distance between the inferior orbital fissure and inferior orbital margin, our findings are comparable to the observations by Kazkayasi et al. [24] in the Turkish population, Przygocka et al. [20] in the Polish population, Nguyen et al. [1] in the United States population. However, Karakas et al. [26] found higher measurements (~32 mm) in the Caucasian population, while Ekambaram et al. [13] observed much lower values (~20 mm) in the South Indian population.

There were some limitations of this study. First, the study has a limited sample size of 90 hemi-skulls, limiting the generalization of its findings. Second, the measurements were made manually and could vary when performed by different assessors. Third, the study is based on the cadaveric specimen obtained from the North Indian population and their actual origin was not confirmed in this study. Lastly, the dimensions could be affected by normal wear of cadaveric specimens, which may not be macroscopically evident.

Nevertheless, the study points towards important findings of heterogeneity of measurements related to infraorbital foramen localization. These findings mean that it may not be feasible to standardize the location of the infraorbital for fine procedures. However, good clinical experience and help from adjuncts line ultrasonography can help in procedures around infraorbital foramen.

## Conclusions

Knowing the relationship between infraorbital foramen and adjacent bony landmarks can help estimate its location. However, our findings suggest that such an estimation is difficult to standardize, considering the wide interindividual variations in the foramen relations. Moreover, these dimensions are further prone to inter-population-related variations, as evident from the published literature. Further research should be performed to investigate the morphometric parameters that locate the infraorbital foramen and are least affected by individual variations in skull morphology. The use of adjuncts like clinical palpation and radiological investigations may help precisely locate infraorbital foramen.
